# Connectivity defines the distinctive anatomy and function of the hand-knob area

**DOI:** 10.1093/braincomms/fcae261

**Published:** 2024-08-13

**Authors:** Ahmad Beyh, Henrietta Howells, Davide Giampiccolo, Daniele Cancemi, Francisco De Santiago Requejo, Salvatore Citro, Hannah Keeble, José Pedro Lavrador, Ranjeev Bhangoo, Keyoumars Ashkan, Flavio Dell’Acqua, Marco Catani, Francesco Vergani

**Affiliations:** NatBrainLab, Institute of Psychiatry, Psychology and Neuroscience, King’s College London, London SE5 8AF, UK; Department of Psychiatry, Brain Health Institute, Rutgers University, Piscataway, NJ 08854, USA; NatBrainLab, Institute of Psychiatry, Psychology and Neuroscience, King’s College London, London SE5 8AF, UK; Department of Clinical and Experimental Epilepsy, UCL Queen Square Institute of Neurology, University College London, London WC1N 3BG, UK; Victor Horsley Department of Neurosurgery, National Hospital for Neurology and Neurosurgery, London WC1N 3BG, UK; Department of Neurosurgery, Institute of Neurosciences, Cleveland Clinic London, London SW1X 7HY, UK; NatBrainLab, Institute of Psychiatry, Psychology and Neuroscience, King’s College London, London SE5 8AF, UK; NatBrainLab, Institute of Psychiatry, Psychology and Neuroscience, King’s College London, London SE5 8AF, UK; IRCCS, SYNLAB, SDN,Naples 80121, Italy; NatBrainLab, Institute of Psychiatry, Psychology and Neuroscience, King’s College London, London SE5 8AF, UK; Neurosurgical Department, King’s College Hospital, London SE5 9RS, UK; Neurosurgical Department, King’s College Hospital, London SE5 9RS, UK; Neurosurgical Department, King’s College Hospital, London SE5 9RS, UK; NatBrainLab, Institute of Psychiatry, Psychology and Neuroscience, King’s College London, London SE5 8AF, UK; IRCCS, SYNLAB, SDN,Naples 80121, Italy; Neurosurgical Department, King’s College Hospital, London SE5 9RS, UK

**Keywords:** motor, TMS, hand knob, connectivity, tumour

## Abstract

Control of the hand muscles during fine digit movements requires a high level of sensorimotor integration, which relies on a complex network of cortical and subcortical hubs. The components of this network have been extensively studied in human and non-human primates, but discrepancies in the findings obtained from different mapping approaches are difficult to interpret. In this study, we defined the cortical and connectional components of the hand motor network in the same cohort of 20 healthy adults and 3 neurosurgical patients. We used multimodal structural magnetic resonance imaging (including T1-weighted imaging and diffusion tractography), as well as functional magnetic resonance imaging and navigated transcranial magnetic stimulation (nTMS). The motor map obtained from nTMS compared favourably with the one obtained from functional magnetic resonance imaging, both of which overlapped well within the ‘hand-knob’ region of the precentral gyrus and in an adjacent region of the postcentral gyrus. nTMS stimulation of the precentral and postcentral gyri led to motor-evoked potentials in the hand muscles in all participants, with more responses recorded from precentral stimulations. We also observed that precentral stimulations tended to produce motor-evoked potentials with shorter latencies and higher amplitudes than postcentral stimulations. Tractography showed that the region of maximum overlap between terminations of precentral–postcentral U-shaped association fibres and somatosensory projection tracts colocalizes with the functional motor maps. The relationships between the functional maps, and between them and the tract terminations, were replicated in the patient cohort. Three main conclusions can be drawn from our study. First, the hand-knob region is a reliable anatomical landmark for the functional localization of fine digit movements. Second, its distinctive shape is determined by the convergence of highly myelinated long projection fibres and short U-fibres. Third, the unique role of the hand-knob area is explained by its direct action on the spinal motoneurons and the access to high-order somatosensory information for the online control of fine movements. This network is more developed in the hand region compared to other body parts of the homunculus motor strip, and it may represent an important target for enhancing motor learning during early development.

## Teaser

Using multimodal brain imaging, Beyh *et al*. show that the ‘hand-knob’ cortical motor area influences spinal motoneurons and accesses high-order somatosensory information through the unique convergence of long and short white matter fibres. Their findings offer insights into cortical motor control and better surgical planning.

## Graphical Abstract

**Graphical Abstract:**
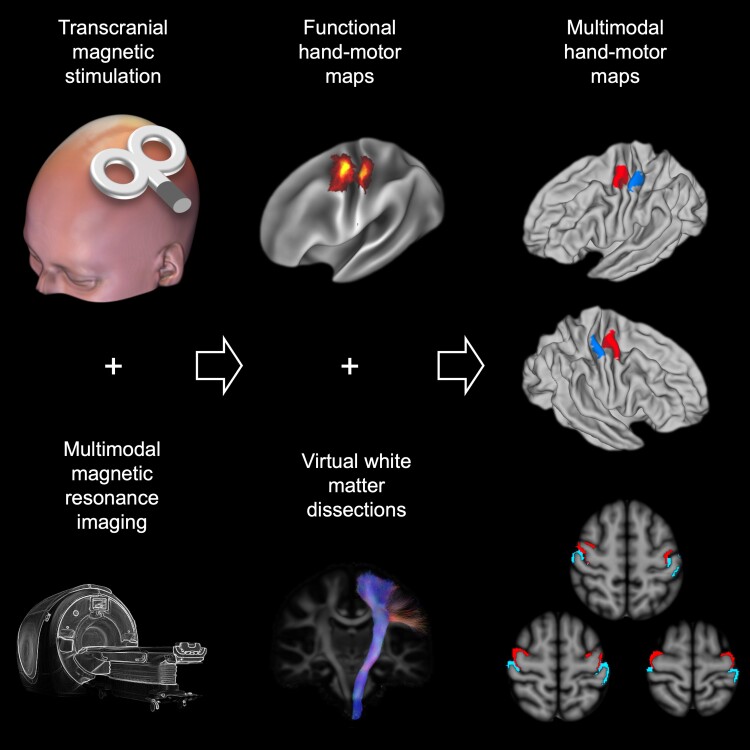

## Introduction

Local association tracts connecting adjacent gyri were first described in early post-mortem studies of connectional anatomy^1-3^ yet have remained understudied to the present day compared with other fibre bundles. These U-shaped tracts are the most superficial of all white matter pathways, travelling beneath the sixth cortical layer.^4^ They are the last to myelinate, leading to speculation that they may be susceptible to plastic changes or compensation and may be useful targets for rehabilitation.^5^ The advent of diffusion tractography has enabled researchers to visualize these tracts non-invasively, confirming the early findings of the classical neuroanatomists, although it is still unclear what role these fibres play in brain function. One intriguing finding has been that the U-fibres connecting primary motor (M1) and somatosensory cortex (S1), which run along the entire extent of the central sulcus, are not homogeneously distributed.^6-8^ Tractography studies have shown that these fibres are concentrated around the distinctive ‘hand-knob’ area,^7,9-11^ indicating that they may play an important role in hand motor control.

Refined independent digit movements are due to the evolution of a corticospinal system conveying motor output mainly from M1, which is synthesized with digit-specific somatosensory and proprioceptive information incoming to S1.^12,13^ Sensory information is rapidly integrated with motor output to generate muscle synergies appropriate for the given motor action.^14-17^ Somatosensory integration occurs at the level of the thalamus; however, studies using muscimol in macaques have indicated that a transient inactivation of S1 causes grasping deficits, suggesting that a direct route may also exist, theorized to be mediated by direct S1 fibres.^18^ Reciprocal connections between primary motor and somatosensory regions have also been identified in non-human primates indicating that M1–S1 U-shaped connections are highly conserved across species.^19-22^

Task-based functional magnetic resonance imaging (fMRI) is the most popular technique available to non-invasively identify cortical regions recruited while the hand performs a motor task, even though it cannot distinguish between regions involved in motor output or sensorimotor integration. Alternatively, single-pulse transcranial magnetic stimulation (TMS) reliably induces motor-evoked potentials (MEPs) in muscles of the hand at rest when applied over M1 with no sensory input. The integration of MRI-navigated TMS (nTMS) approaches have also enabled a more precise mapping of M1 and neighbouring cortices, in particular to produce functional ‘motor maps’ with dense spatial sampling,^23^ clarifying functional differences within the precentral gyrus. Recent evidence has indicated that nTMS has a field of activation that is less than 2 mm wide.^24^ By contrasting these maps identified using fMRI and TMS with tract terminations, it may be possible to examine the relative contributions of different structural connections to the facilitation of motor output and/or sensorimotor integration.

Such an improved understanding of the sensorimotor region is of particular relevance for neurosurgical practice, especially for surgeries near motor eloquent lesions. Functional maps assist surgeons in planning the surgical approach and in stratifying patients according to their risk of developing motor deficits after surgery.^25^ This has important implications in planning early postoperative physiotherapy and improving patients’ understanding during the consent process.^26-30^

We therefore set out to examine how the structural connectivity of primary motor and somatosensory cortex relates to the functional motor maps that can be derived from nTMS and fMRI and its application to neuro-oncological surgical procedures. To this end, we first identify the hand motor map using fMRI and nTMS, as well as the long projection and short association white matter fibres of this region in a healthy cohort. We then compare the various maps obtained within each hemisphere, as well as between the two hemispheres. Finally, we demonstrate the applicability of such a mapping approach in a surgical context.

## Methods

### Subjects

Twenty healthy right-handed adults participated in the study (11 females, mean age = 28 years, SD = 5). Three patients with tumours involving the precentral or postcentral gyrus were also recruited from King’s College Hospital, from a complementary study. Written and informed consent to participate in this research was obtained from all participants and patients, and the Psychiatric, Nursing and Midwifery subcommittee of the College Research Ethics Committee at King’s College London and South Yorkshire Research Ethics Committee approved the study. Healthy participants were excluded if they experienced preterm or difficult birth or any history of neurological or psychiatric disease. Handedness was assessed using the Edinburgh Handedness Inventory.^31^

### MRI data acquisition

#### Structural MRI

Neuroimaging data were acquired using a 3T MR750 MRI scanner (General Electric). Structural T1-weighted (T1w) images were acquired using a 3D FSPGR sequence with the following parameters: 176 axial slices, echo time (TE) = 3.2 ms, repetition time (TR) = 8.2 ms, flip angle = 12°, matrix size = 256 × 256 and field of view (FOV) = 230 × 230 mm^2^, resulting in 0.9 × 0.9 × 0.9 mm^3^ voxels.

#### Functional MRI

Functional MRI data were acquired using a gradient-echo echo-planar imaging (EPI) sequence to detect blood oxygenation level-dependent (BOLD) changes in tissue contrast (TR = 2000 ms, TE = 30 ms, flip angle = 75°, matrix size = 64 × 64 and axial FOV = 211 × 211 mm^2^, resulting in voxel size = 3.3 × 3.3 × 3.3 mm^3^). A single brain volume consisted of 41 axial slices covering the entire brain. Task-related block design fMRI was used, recording repetitive isometric finger abduction with the thumb and finger. Participants were required to tap the little finger or thumb of the left or right hand in response to a visual cue. Each block was 10 s long, with eight fixations lasting 15 s each and a total task duration of 8 min.

#### Diffusion MRI

Diffusion data were acquired using a cardiac-gated single-shot spin-echo EPI sequence and 32-channel head coil (Nova Medical) with the following parameters: 75 axial slices, TE = 80 ms, TR = 5 R-R intervals (approximately 4000 ms), acquisition matrix 128 × 128 and FOV 256 × 256 mm^2^, resulting in 2 × 2 × 2 mm^3^ voxels. Data were collected along 92 diffusion directions with a *b*-value of 2500 s·mm^-2^. A total of 18 non–diffusion-weighted (b0) volumes were collected, 12 with anterior-to-posterior (AP) phase encoding orientation and 6 with posterior-to-anterior (PA) phase polarity for susceptibility distortion correction. A multiband acceleration factor of 3 and an in-plane acceleration factor (ARC) of 2 were used.

### nTMS data acquisition

TMS was delivered using the navigated Nexstim NBS 5.1 system (Nexstim Oy, Helsinki, Finland). The crus of the helix bilaterally, the nasion, and nine more scalp regions were used as cranial landmarks to coregister the patient’s head with their structural T1w image. The maximum stereotactic error allowed by the coregistration software was 2 mm. Electromyography recordings of MEPs from three hand muscles (first dorsal interosseous, abductor pollicis brevis, and abductor digiti minimi) were recorded during the TMS procedure. Monophasic single pulses were delivered through a cooled figure-of-eight coil with an outer diameter of 16.5 cm and inner diameter of 9 cm. The hand-knob region of M1 was identified visually on the Nexstim software using radiological landmarks.^9^ For each participant, stimulation was first delivered across sites on the precentral gyrus to identify a ‘hotspot’ with the highest MEP amplitude. Each hotspot was tested using different coil directions to find the direction causing the highest MEP amplitude. The resting motor threshold (RMT) was determined for the hotspot with the fixed coil direction, using an automated algorithm selecting the highest percentage of stimulator output in 5 out of 10 stimulations.^32^ The coil direction was perpendicular to the central sulcus to achieve maximum field induction. As dense spatial mapping is reputed to offer more reliable information on brain maps,^23^ motor mapping was performed by stimulating with an average distance of 3 mm starting from M1 and progressing more distally in steps of around 3 mm until a line of non-responsive stimulations surrounding the motor area was found.

Only stimulations identified on the precentral or postcentral gyri were considered. MEP amplitude and latency were recorded for all precentral and postcentral stimulation sites. To eliminate any confounds due to autonomous muscle contraction, only sites with MEP amplitudes higher than 50 µV and with latencies between 18 and 30 ms were retained.^33,34^ We compared the MEP profiles of the precentral and postcentral gyri by normalizing to a *Z*-score for each participant. This approach allowed us to account for the expected inter-individual variability in latency and amplitude which vary with bodily anatomy.

### Surgical technique

The patients included in this paper were operated in a single institution. 5-Aminolevulinic acid was used in all patients (oral administration, 20 mg/kg until a maximal dose of 1500 mg, 2 h prior to surgery).

PENTERO 900 Microscope (ZEISS Medical Technology®) equipped with the BLUE 400 filter and Stealth S7 (Medtronic®) were used for surgical resection and intraoperative navigation. Intraoperative neuromonitoring and mapping (IONM) was used during surgery to assess the integrity of transcranial MEPs (tcMEPs), somatosensory-evoked potentials, and direct cortical MEPs. Mapping of motor areas employed the ‘train of five’ technique^35^ both at the cortical and subcortical levels. tcMEPs used twisted needles in the C3, Cz and C4 positions according to the international 10-20 EEG system, and the direct cortical MEPs were recorded from a subdural strip placed under direct vision over M1 (assisted by the anatomical definition of the hand knob and the preoperative mapping). For both cortical and subcortical mapping, the monopolar probe (inomed Medizintechnik GmbH®) was used, and the 1 mm ≈ 1 mA subcortical intraoperative mapping rule^36^ was employed during the projection motor tract mapping. Each positive stimulation site was marked with a numbered tag, a specific motor response was obtained, and the stimulation threshold was recorded. At the end of both cortical and subcortical mappings, an intraoperative photo was taken for documentation purposes. This was used for collocation analysis with the preoperative cortical and subcortical mapping. The surgical resection was limited by the documented positive cortical and subcortical intraoperative stimulation.

### MRI pre-processing and analysis

#### Structural MRI

The T1w image was brain extracted using *optiBET*^37^ and corrected for intensity bias using the *N4 bias correction tool.*^38^ The brain-extracted images were then rigidly aligned with the MNI152 T1w (1 mm) brain template using FSL’s *flirt*^39^ to standardize the head and brain orientation across individuals. The hand-knob region was identified as the omega-shaped contour of the central sulcus as seen on axial slices.^9^

#### Functional MRI

All fMRI data were pre-processed and analysed using FSL’s *FEAT*, version 6.0.^40^ Task-based statistical parametric maps were computed for each condition versus resting baseline at the first level. Activation maps were thresholded using clusters determined by *Z* > 3.1 and were family-wise error corrected using a cluster significance threshold of *P* < 0.001 with *FLAME*.

#### Diffusion MRI

Raw diffusion weighted imaging (DWI) data were first corrected for noise^41^ and Gibbs ringing^42^ artefacts. A magnetic susceptibility field was then calculated using *topup*^43^ based on the pairs of b0 images acquired with opposite phase-encoding. All images were then corrected for motion and eddy current distortions using *eddy*^44^ with outlier slice (signal dropout) replacement,^45^ incorporating the *topup* field into this step.

#### Tractography

StarTrack (https://www.mr-startrack.com/) was used to compute the tractography of the precentral and postcentral projection fibres and the U-fibres that connect the precentral and postcentral gyri. Spherical deconvolution was based on a damped version of the Richardson–Lucy algorithm^46,47^ with the following parameters: fibre response *α* = 1.5, number of iterations = 300, amplitude threshold *η* = 0.0015, and geometric regularization *ν*= 16. This algorithm was chosen as it can resolve crossing fibres in deep white matter regions where projection fibres cross other major association and callosal fibres. A dispersion tractography approach was followed to explore the full profile of the fibre orientation distribution function (fODF) in each voxel. This approach follows the principal fibre orientations indicated by the fODF local maxima but can also follow the directions captured by other vertices of the fODF as they convey information about other local fibre orientations, i.e. they are informed by anatomical fibre dispersion.^47^ Fibre tracking was performed according to the following parameters: minimum hindrance modulated orientational anisotropy (HMOA) threshold = 0.003, number of seeds per voxel = 10, maximum angle threshold = 25°, minimum fibre length = 20 mm and maximum fibre length = 300 mm.

MegaTrack^48^ was used to dissect both the U-fibres and projection fibres. This is a group-level approach that allows the simultaneous virtual dissection of multiple subjects in a common space while maintaining their individual tractography data. Therefore, tensor-based registration was performed to create a study-specific template using TORTOISE.^49,50^ The resulting affine and non-linear transformations were then applied to each subject’s previously computed tractogram to remap all tractograms to the space of the template. All remapped tractograms were concatenated to create a single ‘mega’ tractogram that can be treated as a single subject for virtual dissections. Virtual dissections of the MegaTrack were performed in common space using TrackVis (http://trackvis.org), using criteria laid out in Catani *et al*.^7^ and Catani and Thiebaut de Schotten.^51^

### Analysis of cortical projections

#### Cortical surface reconstruction

A standard FreeSurfer^52^ pipeline was used to identify and reconstruct the grey–white matter boundary and the external, pial boundary of each subject’s pre-processed T1w brain image. Additional steps using tools from Connectome Workbench (https://www.humanconnectome.org/software) were applied to remap each subject’s surface to a common space, the ‘32k_FS_LR’ template. This template offers vertex-wise matching across subjects while maintaining the native surface anatomy and morphology of each subject. The midthickness, inflated and very inflated surfaces were also produced at this stage.

#### TMS cortical maps

A recent analysis of the spread of the induced field using an 8-shaped coil showed that only the central region under the coil centre is stimulated using single-pulse TMS.^24^ Additionally, each TMS stimulation performed during a mapping session is associated with two sets of coordinates: one set represents the location of the centre of the TMS coil which lies just outside the skull, while the other corresponds to the location of the maximal intensity of the induced field (EFmax) and is based on a spherical head model. The latter point can fall within the cortical ribbon or within the outermost layers of white matter, depending on multiple factors such as hair volume, skull thickness, cortical thickness and the distance between the skull and the cortical patch in question. In cases where this skull-to-cortex distance is large, the EFmax point might lie just above the cortical surface. Due to this variability, neither of these coordinates can be used independently to accurately define the cortical location targeted by the TMS stimulation.

To overcome this challenge, for every TMS stimulation, a line connecting the coil centre to the EFmax point was defined. Then, using a ray-triangle intersection algorithm, the face (triangle) of the pial mesh closest to the coil and intersected by this line was identified. Finally, the vertices connected to this face were selected as targets of that specific TMS stimulation. This effectively allowed us to locate the most probable target of each TMS stimulation on the cortical surface. Of course, this resulted in patchy cortical TMS representations as each stimulation is associated with a focal region of the cortex confined to three mesh vertices (one triangular face). To derive contiguous cortical masks that represent the entire cortical patch where TMS stimulation gave rise to hand motor responses, the vertices that were previously selected were used to define a contour, and any vertices within this contour were included in the final mask. All steps related to the TMS cortical projection were performed using in-house functions written in MATLAB (The MathWorks).

#### fMRI cortical maps

To display the fMRI-based activation maps on the cortical surface, the midthickness mesh was used. This surface lies between the grey–white matter boundary (deepest) and the pial boundary (shallowest) of the cortex and offers the highest level of intersection with voxels representing grey matter, with minimal bias towards the surrounding cerebrospinal fluid-filled or white matter voxels. Therefore, each vertex from the midthickness surface was used to perform a trilinear interpolation of the voxel-wise fMRI maps produced previously to convert these maps to a vertex-wise representation. This step was performed using in-house functions written in MATLAB (The MathWorks).

#### Tractography cortical maps

Most streamlines reconstructed with tractography terminate around the grey–white boundary due to the anisotropy thresholds imposed during tracking, but they do so at varying distances from this boundary. In certain areas, high anisotropy can be found even within the deeper layers of cortex, especially due to partial volume effects observed at the typical 2 mm voxel size used in DWI acquisitions. Therefore, streamlines might terminate underneath, at or above the grey–white boundary. To account for this variability while estimating the cortical projections of streamlines, we adopted an approach similar to the one described in Beyh *et al*.^53^: for every streamline, each of its two end points was used to define the centre of a sphere of 3 mm radius; any vertices of the white matter surface that fell within the sphere were included as possible cortical targets of that streamline. This method was implemented in custom functions written in MATLAB (The MathWorks).

#### Group-level cortical representations

The previously described cortical maps for each modality were created in the native anatomical space of each subject. Therefore, to create group overlap maps for a given modality, each subject’s surface data were first converted to a binary mask, then all subjects’ binary masks were summed to create the final overlap maps. To focus on the common area of overlap between TMS and fMRI, we produced group maps showing cortical regions where 30% or more of participants showed a response.

#### Statistical analysis

Where applicable, the tests used for statistical comparisons between gyri or hemispheres are reported along with their details in the Results section.

To quantify the overlap between the two hemispheric cortical maps of each modality (TMS, fMRI, and tractography projections), a dice coefficient was computed as follows:


(1) $$\begin{array}{c} d=2\times \frac{\mathrm{are}a_{L\cap R}}{\mathrm{are}a_{L\cup R}} \end{array}$$

where area_L_ and area_R_ are the surface area of the given modality’s cortical mask in the left and right hemispheres, respectively. Given the matched surface representation between the two hemispheres owing to the use of the ‘FS_LR_32k’ surface template, data from the left and right hemispheres could be directly compared.

## Results

### Functional mapping

**Figure 1:**
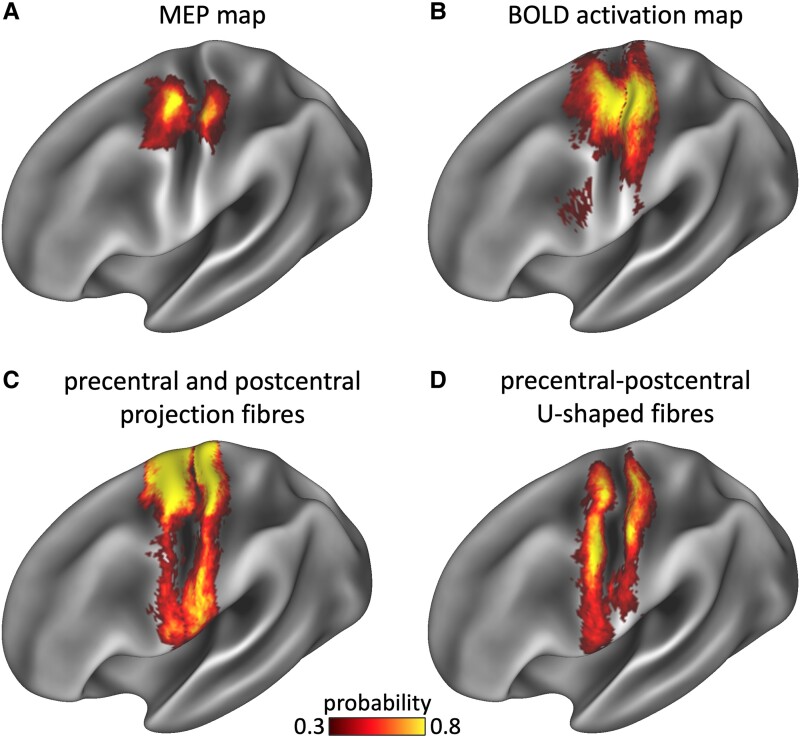
**Group functional and tractography cortical maps.** All panels show a left hemisphere representation of group probability maps. (**A**) Cortical TMS stimulation loci that led to MEPs in hand muscles. (**B**) Observed fMRI activations during the finger tapping task. (**C**) Cortical projections of the precentral and postcentral projection fibres. (**D**) Cortical projections of the U-fibres connecting precentral and postcentral gyri.

We used nTMS to locate hand motor responses in the precentral and postcentral gyri in a control cohort. We found such responses in both gyri of the left hemisphere in all participants. In the right hemisphere, we found such responses in all participants, except two who did not have them in the postcentral gyrus. The ‘hand-knob’ region was contained within the activation map in all subjects (Fig. 1A). The TMS RMT was on average 35.6 ± 8.7% in the dominant hemisphere and 35.6 ± 8.4% in the non-dominant hemisphere. The number of stimulation sites in the precentral gyrus was on average 70% higher than in the postcentral gyrus, and this difference was statistically significant in the left [*t*(18) = 1.76, *P* = 0.048, one-tailed] and the right hemispheres [*t*(18) = 3.05, *P* = 0.003, one-tailed]. On average, 38 ± 19 sites were identified in the left hemisphere and 41 ± 15 in the right hemisphere. There was no significant difference in the number of sites between hemispheres for the precentral gyrus [*t*(18) = -0.79, *P* = 0.779, one-tailed, *n.s.*] nor the postcentral gyrus [*t*(18) = 0.17, *P* = 0.432, one-tailed, *n.s.*].

**Figure 2:**
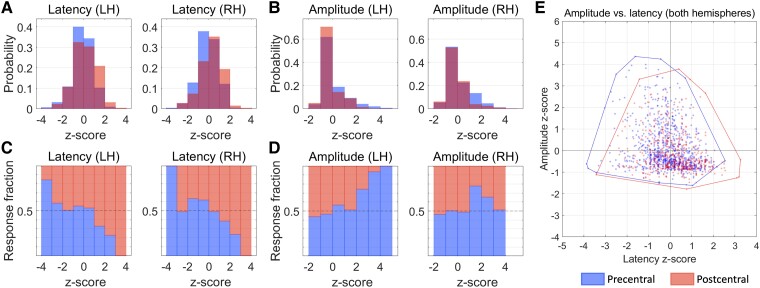
**Latencies and amplitudes of MEPs obtained through nTMS.** The standardized (*Z*-score) latencies and amplitudes of MEPs obtained through nTMS applied to the precentral and postcentral gyri are compared in these plots. These scores were calculated separately for each participant to account for expected individual differences in latencies and amplitudes. In the first row, the probability distribution for latencies (**A**) and amplitudes (**B**) is shown for each hemisphere. In **C** and **D**, the proportion of precentral and postcentral MEPs are directly compared for each *Z*-score range. On average, latencies of precentral MEPs were significantly shorter than those of postcentral MEPs in both hemispheres [left: *t*(19) = -3.5, *P* < 0.001; right: *t*(19) = -3.36, *P* < 0.001; Table 1]. MEP amplitudes were also significantly higher in the precentral gyrus in both hemispheres [left: *t*(19) = 2.34, *P* = 0.019; right: *t*(19) = 2.91, *P* = 0.004; Table 1]. **E** shows a boundary plot of the pooled data from all subjects and both hemispheres. The overall observation from these plots is that, although the latencies and amplitudes of MEPs that follow stimulation of the precentral and postcentral gyri overlap substantially, the stimulation sites of the MEPs with the shortest latencies and largest amplitudes are more frequently localized in the precentral gyrus. LH: left hemisphere; RH: right hemisphere.

We compared the standardized (*Z*-score) amplitudes and latencies of the MEPs in the precentral and postcentral gyri. On average, latencies of precentral MEPs were significantly shorter than those of postcentral MEPs in both hemispheres [left: *t*(19) = -3.5, *P* < 0.001; right: *t*(19) = -3.36, *P* < 0.001; Table 1; Fig. 2A and C]. MEP amplitudes were also significantly higher in the precentral gyrus in both hemispheres [left: *t*(19) = 2.34, *P* = 0.019; right: *t*(19) = 2.91, *P* = 0.004; Table 1; Fig. 2B and D]. Although MEPs with short latencies and high amplitudes were more prevalent in the precentral gyrus, most recorded MEPs were similarly distributed in the precentral and postcentral gyri (Fig. 2E).

**Table 1:** Pre- versus postcentral functional mapping results from MEP and BOLD

Measure	Precentral *Z*-score	Postcentral *Z*-score	*t*	*P*
Latency (LH)	−0.11 ± 0.94	0.17 ± 1.03	−3.50	<0.001**
Latency (RH)	−0.09 ± 0.96	0.17 ± 1.02	−3.36	<0.001**
Amplitude (LH)	0.07 ± 1.05	−0.11 ± 0.87	2.34	0.019*
Amplitude (RH)	0.08 ± 1.00	−0.14 ± 0.94	2.91	0.004*
BOLD (LH)	6.11 ± 1.19	6.03 ± 1.00	0.54	0.590
BOLD (RH)	6.12 ± 1.19	6.04 ± 1.00	0.53	0.599

LH, left hemisphere; RH, right hemisphere. *P < 0.05, **P < 0.001.

BOLD activation in the sensorimotor cortex was observed in all subjects during the finger tapping task (Fig. 1B). Unlike the TMS results, fMRI *Z*-scores of the precentral gyrus were not statistically different from those of the postcentral gyrus in either hemisphere [left: *t*(19) = 0.54, *P* = 0.590, *n.s.*; right: *t*(19) = 0.79, *P* = 0.437, *n.s.*; Table 1]. The hand-knob region of the primary motor cortex was contained within the activation map in all participants (Fig. 1B).

### Diffusion tractography

**Figure 3:**
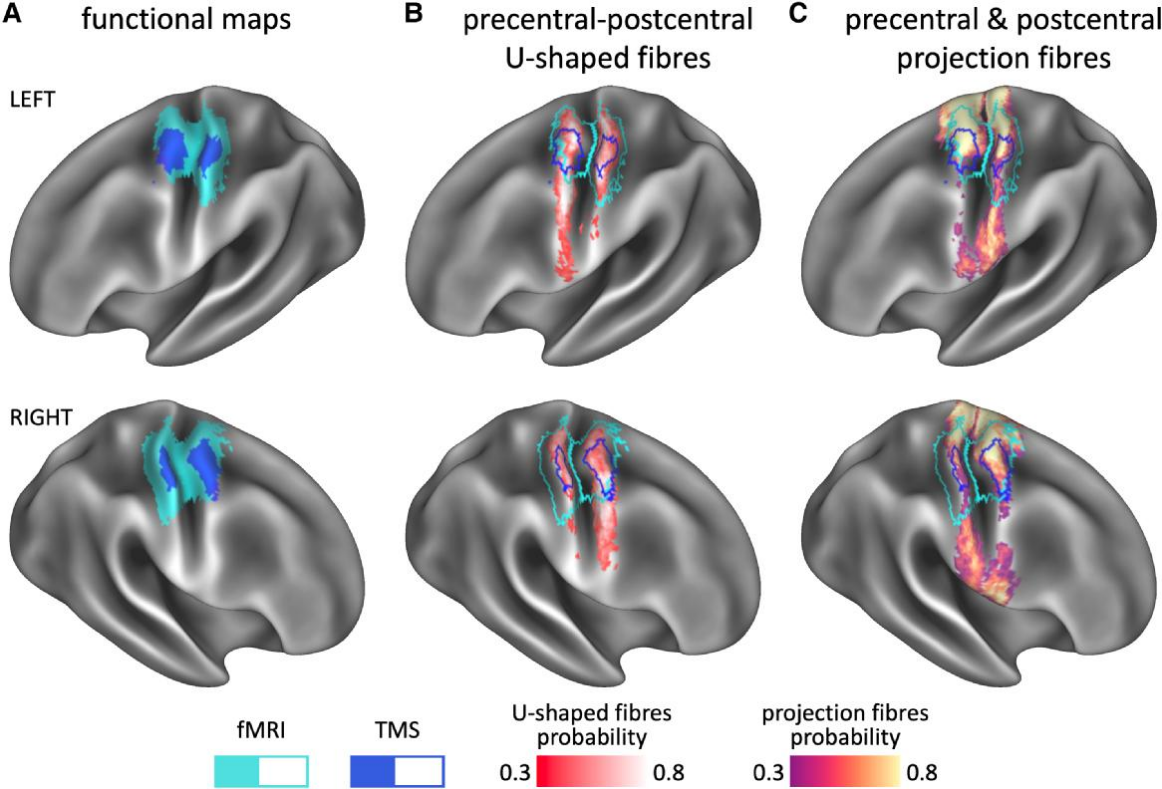
**Multimodal overlap on the cortical surface.** The four different maps of interest are compared in each hemisphere. In **A**, nTMS and fMRI maps are overlaid on the cortical surface for direct comparison. Unlike the TMS scores (Table 1 and Fig. 2), fMRI *Z*-scores of the precentral gyrus were not statistically different from those of the postcentral gyrus in either hemisphere [left: *t*(19) = 0.54, *P* = 0.590, *n.s.*; right: *t*(19) = 0.79, *P* = 0.437, *n.s.*; Table 1]. These functional maps are overlaid as contours on top of the projections of the precentral–postcentral U-fibres in **B** and those of the projection fibres in **C**. Percentage overlap scores for these displays are reported in Table 3.

Projection fibres extending between the precentral gyrus and brainstem and between the brainstem and postcentral gyrus were traced in all participants (Fig. 2A). U-fibres running between the precentral and postcentral gyri along the entire extent of the central sulcus were also generated (Fig. 2B). Statistical comparisons of the left and right projection tracts and U-fibres based on *t*-tests are reported in Table 2. On average, the projection tracts had slightly larger volume and lower HMOA (fibre density) in the left hemisphere compared to the right. These results indicate that the left corticospinal tract (CST) was larger and had potentially larger axons in the left hemisphere, likely due to the right-hand dominance of the participants in our sample. No hemispheric differences were found in volume or HMOA of the U-fibres. The projection fibres and U-fibres overlapped within the hand-knob bulge of the precentral gyrus and an adjacent region of the postcentral gyrus (Fig. 3).

**Table 2:** Macro- and microstructural hemispheric tract comparisons

Measure	Left hemisphere	Right hemisphere	*t*	*P*
Projection fibres volume (mL)	25.78 ± 3.94	23.89 ± 2.74	−2.55	0.019*
U-fibres volume (mL)	12.76 ± 3.45	13.58 ± 3.22	1.50	0.149
Projection fibres HMOA	0.0158 ± 0.0011	0.0165 ± 0.0012	3.47	0.003*
U-fibres HMOA	0.0090 ± 0.0006	0.0087 ± 0.0008	−1.65	0.116

*P < 0.05.

### Correspondence between structural and functional methods

Figure 3 shows the overlap between the fMRI and nTMS maps and tractography projections of the precentral–postcentral projection fibres and U-fibres. The nTMS motor map of the hand is fully contained within the fMRI sensorimotor map, but the latter extends to cover a larger cortical area both medially and laterally. The region where the two coincide falls within the ‘hand-knob’ zone of the precentral gyrus and an adjacent region in the postcentral gyrus. As the projection and U-fibres were dissected without limiting their extent along the central sulcus, their cortical projections extend to a large area of both the precentral and postcentral gyri, larger than the area covered by either nTMS or fMRI. Both functional maps overlap with the tractography projections of the projection and U-fibres in the hand region, but not to the same extent.

Table 3 shows the proportion of the nTMS and fMRI maps connected to the precentral–postcentral U-fibres and projection fibres. The projection fibres covered a large proportion of both the fMRI and nTMS maps, particularly in the precentral gyrus. In comparison, the U-fibres had a better overlap with the functional maps in the postcentral gyrus. Table 3 conversely shows the proportion of the tractography projections of the U-fibres and projection fibres covered by the nTMS and fMRI maps. Both functional maps covered a larger fraction of the precentral projection fibres than postcentral projections, and the fMRI map, which is larger in size, covered a larger proportion of the projection fibres than the nTMS map (in both hemispheres). Conversely, both the nTMS and fMRI maps covered a larger fraction of the cortical projections of the U-fibres in the postcentral gyrus compared with the precentral gyrus in both hemispheres. Moreover, there was a particularly good overlap between the fMRI map and the U-fibre projections in the postcentral gyrus: more than 90% of the postcentral U-fibre projections were covered by the fMRI map (Table 3).

**Table 3:** Overlap between functional maps and tractography projections

Proportion of functional maps reached by tractography projections				
Functional map	% covered by motor projections		% covered by U-fibre projections	
	Precentral	Postcentral	Precentral	Postcentral
TMS (LH)	77%	81%	57%	96%
TMS (RH)	89%	77%	80%	84%
fMRI (LH)	78%	63%	52%	63%
fMRI (RH)	75%	44%	48%	44%

Proportion of tractography projections covered by functional maps				
Tract	% covered by nTMS map		% covered by fMRI map	
	Precentral	Postcentral	Precentral	Postcentral
Projection fibres (LH)	22%	13%	47%	42%
Projection fibres (RH)	19%	11%	43%	35%
U-fibres (LH)	24%	33%	46%	90%
U-fibres (RH)	31%	31%	50%	92%

LH, left hemisphere; RH, right hemisphere.

### Hemispheric differences

**Figure 4:**
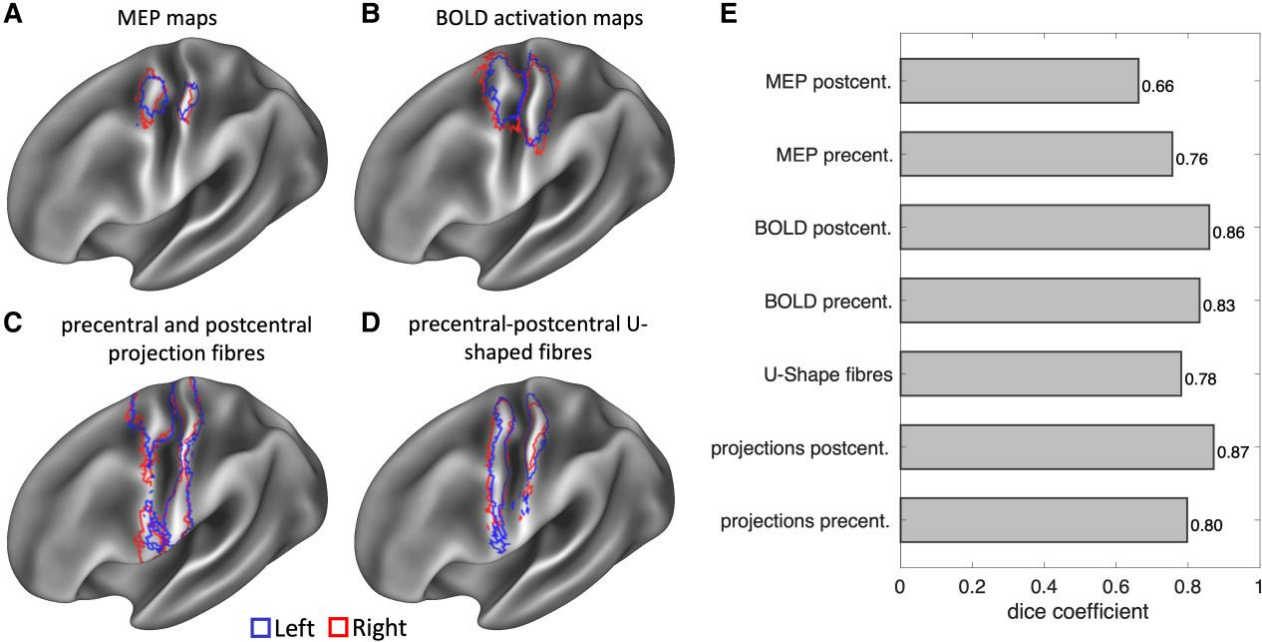
**Interhemispheric comparisons.** In panels **A–D**, the cortical maps of the left (blue) and right (red) hemispheres are overlaid on a left hemisphere cortical representation to visualize their correspondence. **E** shows the Dice coefficients of the same comparisons.

We used Dice coefficients to compare the cortical locations of the functional (nTMS and fMRI) and structural (cortical projections of motor projection fibres and U-fibres) cortical maps between the two hemispheres (Fig. 4). Overall, we found a high level of interhemispheric agreement in the spatial location and extent of each cortical map, with Dice coefficients ranging between 0.66 and 0.87.

We found a high spatial agreement between the left and right nTMS motor maps, indicating that MEPs in the contralateral hand could be identified when stimulating an anatomically conserved territory across the precentral and postcentral gyri in the left and right hemispheres. The fMRI sensorimotor maps also incorporated the same region in the right and left hemispheres, although the map was slightly larger in the right hemisphere. Examination of the structural maps revealed that the projection fibres of the precentral and postcentral gyri and the U-fibres were also highly similar across hemispheres.

### Surgical cases

**Figure 5:**
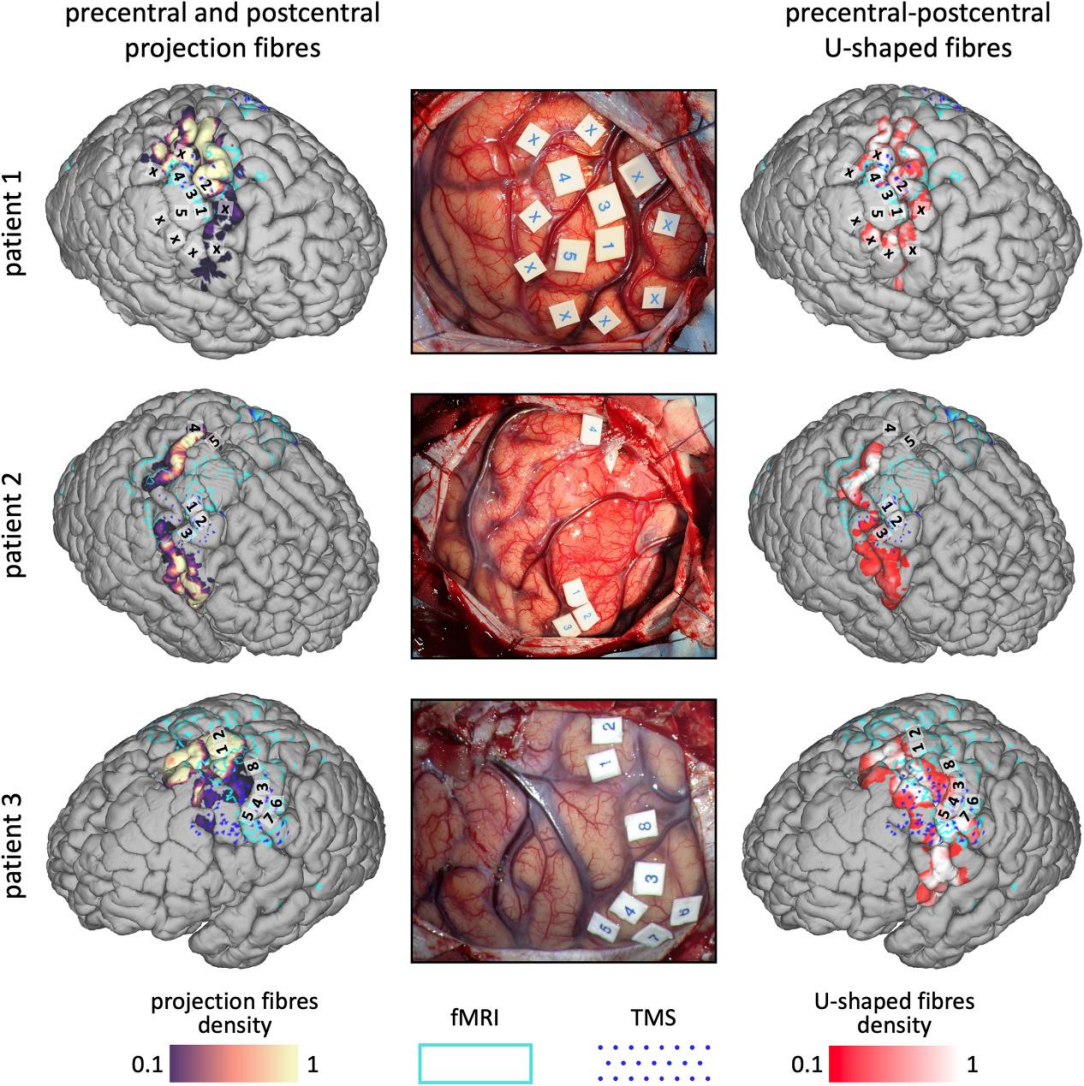
**Clinical examples of the integration of the structural and functional maps with intraoperative neuromonitoring data.** We assessed the relationship between functional nTMS and fMRI maps and tractography cortical projections in three patients who underwent tumour resection surgery. For each patient, the left panel highlights the relationship between the cortical termination of the somatosensory projection tracts (purple–yellow heatmaps), the nTMS (dark blue dots) and fMRI (light blue contour) maps, while the right panel does the same for the termination of the U-fibres (red–white heatmaps). The middle panel shows an intraoperative view of the exposed brain with the locations of the intraoperative cortical stimulations that were performed. These locations are also identified and numbered on the cortical surface reconstructions. Patient 1 tags: 1, 2 and 3—hand and forearm; 4 and 5—hand; x—no response. Patient 2 tags: 1, 2 and 3—hand and forearm; 4 and 5—foot and leg. Patient 3 tags: 1 and 2—lower limb and foot; 3, 4, 5, 6 and 7—hand and forearm; 8: hand only.

We assessed the feasibility of our multimodal approach, combining nTMS, fMRI, and tractography, in three patients presenting with gliomas in eloquent motor areas. In each case, we assessed the correspondence of the nTMS and fMRI functional maps with the tractography projections of the motor projection fibres and precentral–postcentral U-fibres and made a qualitative comparison with intraoperative mapping data (Fig. 5).

Patient 1 was a 48-year-old female who presented with headache. Imaging revealed a right hemisphere tumour in the hand region of the postcentral gyrus. The RMT of the hand was 46% (E-field strength of 79 V/m) in the right hemisphere and 38% (64 V/m) in the left. The intraoperative motor stimulation threshold was 5 mA, and positive cortical response was elicited at the level of the hand knob. Histology confirmed a WHO Grade 2, IDH1-positive astrocytoma.

Patient 2 was a 53-year-old male who presented with focal seizures with subsequent left-sided weakness and slurred speech. Imaging revealed a tumour in the right precentral gyrus. The RMT of the hand was 52% (111 V/m) in the right hemisphere and 22% (50 V/m) in the left. The intraoperative motor stimulation threshold was 10 mA, and positive responses were obtained from the foot and hand areas. Histology revealed a WHO Grade 3 oligodendroglioma, IDH1 positive, 1p/19q co-deleted.

Patient 3 was a 57-year-old male who presented with sudden onset right arm paraesthesia and tingling, suggestive of sensory seizures. Imaging revealed a mass in the left postcentral gyrus near the hand region. The RMT of the hand was 35% (64 V/m) in the left hemisphere and 28% (44 V/m) in the right. The intraoperative motor stimulation threshold was 5 mA, with motor responses elicited from the foot and arm/hand areas. Histology revealed a WHO Grade 3 oligodendroglioma, IDH1 positive, 1p/19q co-deleted.

In all patients, nTMS sites tended to be contained within the fMRI sensorimotor map (Fig. 5). In addition, the colocation of the functional maps with the cortical projections of the motor projection fibres and U-fibres was preserved around the tumour but not within the cortex immediately above it. The intraoperative responses obtained with direct electrical stimulation (DES) using the ‘train of five’^54^ technique in all cases colocalized with positive nTMS responses (within a 3 mm radius) and were contained within the fMRI sensory-motor maps.

## Discussion

Linking structural connectivity of the primary sensorimotor cortex with functionally localized regions is crucial to better understand the detailed cortical and subcortical anatomy involved in hand motor control. We explored this by creating maps of the regions with greatest consistency across subjects using structural (diffusion tractography) and functional (fMRI and nTMS) imaging techniques. Regions of both the precentral and postcentral gyri involved in motor output (MEPs evoked using nTMS) corresponded anatomically with white matter projections from these regions towards the brain stem. We further show that common regions recruited during a sensorimotor finger tapping fMRI task do not only correspond with terminations of precentral and postcentral projection fibres, but also with U-fibres that connect the two gyri, and that in fact fMRI showed better correspondence with U-fibres than corticospinal projections. These regions were highly conserved between hemispheres. Our results indicate that the precentral and postcentral cortices operate as a highly integrated unit and that U-fibres may provide a crucial highway for sensorimotor information flow between the two gyri, along with projection fibres as its main afferent and efferent connections. This supports the early ideas of Hughlings Jackson^55,56^ and Horsley.^57^

Although they are both involved in sensorimotor processing, the precentral and postcentral gyri have different cytoarchitectural characteristics^58,59^ and functional characteristics as demonstrated by nTMS and fMRI. In our study, we evoked MEPs in hand muscles from the postcentral gyrus as well as the precentral gyrus in all participants, which is not commonly reported. Woolsey^60^ reported this in macaques, as did Penfield and Boldrey^61^ in humans, showing that 80% of motor responses occurred in precentral regions and 20% in postcentral regions, with the inverse proportions for sensory responses. Similar results have been reported in a modern revisitation of the Penfield motor homunculus.^29^ We also show that precentral gyrus MEPs had a higher amplitude than the postcentral gyrus and that postcentral gyrus MEPs had longer latencies. We propose two possible hypotheses to explain this: the first concerns the involvement of monosynaptic corticomotoneurons whose origin is in the precentral/postcentral gyrus complex; the second is related to the U-fibres that connect the precentral and postcentral gyri.

Monosynaptic connections to motoneurons have been identified in both pre- and postcentral cortices^62^ and are related to fine control of movement, rather than movement per se, and are more commonly found in humans and great apes than in other species.^13^ These corticomotoneurons belong to the so-called new M1, located on the caudal portion of M1 and extending into the depth of the central sulcus beneath the crowns of the precentral and postcentral gyri. The new M1 is distinct from the old M1, which is located on the rostral part of M1 and is predominantly formed by neurons that project to interneurons within the intermediate part of the spinal cord.^63^ It is therefore possible that the motor responses obtained from the pre- and postcentral gyri in our study are due to stimulation of fibres of the new M1, which is anatomically ‘embedded’ beneath the two gyri. However, we recorded longer latencies for MEPs elicited in the postcentral gyrus (*P* < 0.001), which raises the possibility that there may have been indirect stimulation of the precentral gyrus via the U-fibres of the central sulcus. Interestingly, the two hypotheses point in the same general direction, as both monosynaptic corticomotoneurons^13^ and U-fibres originating from the postcentral gyrus seem to be related to fine motor control. The latter observation is supported by the work of Sasaki *et al.*^64^ who showed that primates were still able to grasp with independent fingers after corticomotoneuronal lesions in the spinal cord, although with important deficits in hand pre-shaping as well as in control over the force between the fingers. This may support the idea that the elicited postcentral MEPs are a product of targeted stimulation of extra-precentral corticomotoneuronal fibres.

The pre- and postcentral gyri are integrated in this anatomical and functional unit by the U-fibres. By tracing all fibres extending between the precentral and postcentral gyri, we observed clear discrepancies in the distribution of their cortical projections along each gyrus, but these fibres most consistently connected cortical regions surrounding the most prominent bulge in the ‘hand-knob’ region.^9^ Anatomical studies have reported that U-fibres are concentrated in the hand-knob region,^7,65^ and our results demonstrate that they correspond functionally with regions involved in hand motor function, most notably in the left hemisphere (in right-handed participants). The projections of these fibres were consistently distributed across a relatively wide area of the precentral gyrus but were more concentrated around the hand knob in the postcentral gyrus. Notably, the crown of the precentral gyrus is regarded to be premotor (dPM) rather than primary motor cortex (M1), the latter being buried within the central sulcus.^59^ TMS is regarded to activate a caudal part of dPM which then propagates through intracortical circuits to M1.^66,67^ In this case, the fibres visualized here that extend subcortically are corticospinal but may not be corticomotoneuronal in type.

We also show that local U-fibres connect the posterior side of the crown of the precentral gyrus with the anterior crown of the postcentral gyrus. This is in line with a study by Viganò *et al*.^16^ in three tumour patients, who showed that precentral–postcentral local fibres occupy a distinct territory of the precentral gyrus compared with U-fibres connecting the precentral and more anterior premotor regions. Here, they applied direct electrical stimulation at the same threshold and showed that stimulating posterior territories led to higher amplitude motor responses compared with anterior territory stimulation. As pyramidal fibres are distributed along the anterior bank of the central sulcus, this may indicate that precentral–postcentral fibres are involved in the final stages of motor processing. Interestingly, short U-fibres projecting between the postcentral and posterior parietal regions also appear to be distributed along the posterior side of postcentral gyrus.^68^ Whether this pattern is a genuine anatomical finding or a methodological artefact of tractography is yet to be confirmed.

The clinical role of damage to the U-fibres in patients is not properly understood. In recent years, few reports in the literature described the feasibility and outcome of surgery for tumours involving the primary motor cortex, whether with asleep or awake techniques.^69,70^ While the emphasis in these clinical reports is on avoiding hemiplegia, U-fibres connecting primary and sensory areas are thought to play a more subtle role in the execution of fine digit movement, as mentioned above. Experiments in primates demonstrated that pharmacological inactivation of the primary sensory cortex causes deficits in precision grasping, most likely through inhibition of the direct sensory feedback provided by the U-fibres.^7,18^ Similar evidence of a direct modulation between sensory and motor areas through a cortico-cortical pathway has been recently reported using dual-site TMS; in this experimental setting, stimulation of S1 attenuated M1 excitability (S1–M1 inhibition) during precision and power grip testing.^71^

The crown of the precentral gyrus that, as mentioned above, belongs to the premotor cortex (dPM) is also connected via U-fibres to the supplementary motor area (SMA). The SMA is a major hub in the motor network, being involved in the planning (pre-SMA) and direct execution (SMA-proper) of complex movements, such as reaching and grasping.^72,73^ Mapping of the SMA has been performed in humans, most frequently with DES, in patients undergoing surgical resection of tumours affecting this region.^74^ These studies demonstrated important properties of the SMA, such as its somatotopy^75^ and its role in neuroplasticity.^76^ More recently, TMS mapping studies of the SMA have clarified the role of its connectivity in action preparation^77^ and the role of non-primary motor areas in patients with brain tumours.^78^ Future studies are therefore needed to investigate the interaction between the SMA and the primary hand-knob area in the organization and execution of motor task and in their relative contribution to motor cognition.

The analysis of the interhemispheric differences with the dice coefficients did not show substantial differences between the two hemispheres apart from a larger representation of the fMRI maps in the right hemisphere. Previous studies^79,80^ reported significant differences between dominant and non-dominant motor activation. Recent data suggest that the individual finger representation in the somatosensory cortex does not seem to be significantly different between the dominant and non-dominant hemisphere.^81^ A possible explanation for this lies in the complexity of the tasks assessed, as the differences are more pronounced in complex motor activities due to the involvement of both dorsal premotor and parietal areas,^82^ whereas the task used here was basic individual finger tapping. This hypothesis is supported by a larger involvement of the associative areas of the non-dominant hemisphere when subjects perform motor tasks with their non-dominant hand.^83^

fMRI and nTMS seem to explore response characteristics in different ways, as previous literature supported a more posterior location of the centre of gravity of motor functional areas defined with fMRI compared with nTMS mapping.^84,85^ This finding is quite robust, and the results were independent of the task or the considered response characteristics,^84^ or the relatively lower spatial specificity of the gradient-echo BOLD fMRI sequence.^85^ In addition, a few studies compared the results of nTMS versus fMRI motor mapping, comparing them with DES in patients having surgery for tumours in eloquent motor area, and again found similar results, with a more posterior activation maximum obtained by fMRI compared to nTMS.^86-88^ A possible explanation may lie in the different types of muscular activity recruited by the two techniques—‘active’ fMRI mapping versus ‘passive’ nTMS mapping—even though it is hypothesized that active nTMS mapping will further increase the distances between the fMRI and nTMS results based on preliminary data obtained from low-level nTMS mapping.^89,90^ An incomplete understanding of nTMS physiology (I-wave activation and motor threshold versus D-wave activation at suprathreshold stimulation), and its interaction with CST fibres (more likely interaction with the anterior fibres leading to a forward shift of the centre of gravity) may also contribute to these observed differences.^85,91^ Despite our efforts to produce accurate nTMS-derived motor maps, the exact localization of nTMS stimulation sites on the cortical surface remains challenging (e.g. they depend on the chosen head model among other factors), and this may have unintended effects on the final maps. Finally, it is worth noting that the fMRI signal measures both the motor and sensory signals in the pre- and postcentral gyri, while nTMS does not measure the sensory component; this fundamental difference may explain the larger observed difference between the two techniques in the postcentral gyrus.

From a clinical perspective, the combined use of nTMS, fMRI, and tractography provided an accurate preoperative mapping for lesions close to motor areas. The data provided from this study have particular relevance when preoperative stratification risk scores are considered.^25,28,92^ The involvement or distance to an fMRI or nTMS map may have a different impact on the risk of developing postoperative deficits, given the differences in the overlap with the projection and U-fibres as described. On the other hand, surgical planning can be optimized by taking into consideration the origin of the functional maps, particularly to assist the use of intraoperative neuromonitoring (IONM). In Fig. 5, we provide clinical examples of patients diagnosed with highly motor eloquent tumours operated using an integrated model of these three preoperative modalities and IONM. Although limited to a sample of three cases, we illustrate the feasibility of obtaining an integrated pre-surgical mapping in patients. The detailed information thus obtained is helpful in the process of informed consent and offers a better understanding of the risks involved and the challenges in operating in motor eloquent areas.^28^

## Conclusion

Three main conclusions can be drawn from our study. First, the hand-knob region is a reliable anatomical landmark for functional localization of fine digit movements. Second, its distinctive shape is determined by the convergence of highly myelinated long projection and short U-fibres. Third, the unique role of the hand-knob area is explained by its direct action on the spinal motoneurons and the access to high-order somatosensory information for the online control of fine movements. This network is more developed in the hand region compared to other body parts of the homunculus motor strip, and it may represent an important target for enhancing motor learning during early development and recovery after brain damage.

## Data Availability

The hand motor map, as defined by the overlap between the nTMS and fMRI maps from this study, can be downloaded as a voxel mask in MNI152 1 mm space and as a cortical surface mask 32k_FS_LR space, through OSF (https://osf.io/wgm4j).
